# HD-tDCS of primary and higher-order motor cortex affects action word processing

**DOI:** 10.3389/fnhum.2022.959455

**Published:** 2022-09-29

**Authors:** Karim Johari, Nicholas Riccardi, Svetlana Malyutina, Mirage Modi, Rutvik H. Desai

**Affiliations:** ^1^Human Neurophysiology & Neuromodulation Lab, Department of Communication Sciences and Disorders, Louisiana State University, Baton Rouge, LA, United States; ^2^Department of Psychology, University of South Carolina, Columbia, SC, United States; ^3^Center for Language and Brain, HSE University, Moscow, Russia; ^4^Weinberg College of Arts and Sciences, Northwestern University, Evanston, IL, United States; ^5^Institute for Mind and Brain, University of South Carolina, Columbia, SC, United States

**Keywords:** lexical semantics, nouns, verbs, embodied cognition, motor cortex, inferior parietal lobe, HD-tDCS

## Abstract

The contribution of action-perception systems of the brain to lexical semantics remains controversial. Here, we used high-definition transcranial direct current stimulation (HD-tDCS) in healthy adults to examine the role of primary (left hand motor area; HMA) and higher-order (left anterior inferior parietal lobe; aIPL) action areas in action-related word processing (action verbs and manipulable nouns) compared to non-action-related control words (non-action verbs and non-manipulable nouns). We investigated stimulation-related effects at three levels of semantic processing: subliminal, implicit, and explicit. Broadly, we found that stimulation of HMA and aIPL resulted in relative facilitation of action-related language processing compared to non-action. HMA stimulation facilitated action verb processing in subliminal and implicit task contexts, suggesting that HMA helps represent action verbs even in semantically shallow tasks. HMA stimulation also facilitated manipulable noun comprehension in an explicit semantic task, suggesting that HMA contributes to manipulable noun comprehension when semantic demands are high. aIPL stimulation facilitated both manipulable noun and action verb processing during an implicit task. We suggest that both HMA and aIPL play a functional role in action semantics. HMA plays a general role in the semantics of actions and manipulable objects, while aIPL is important only when visuo-motor coordination is required for the action.

## Introduction

Multiple brain areas contribute to the representation of lexical semantic knowledge. Across many types of concepts, lateral and medial temporal cortices, as well as the inferior parietal lobe, are important for semantic processing (Binder et al., 2009; Binder and Desai, 2011; Desai and Riccardi, 2021). However, the nature and level of contribution of distributed action-perception systems to semantic representation remains controversial (Mahon and Hickok, 2016). Some evidence suggests that conceptual processing of action-related words such as motor verbs (e.g., *to kick*) and manipulable nouns (e.g., *the pencil*) partially rely on neuroanatomical structures that are involved in movement production, planning, and control. For example, neuroimaging studies have shown that action-related word processing is associated with increased activity in areas such as the primary motor cortex (M1) and anterior inferior parietal lobe (aIPL), which subserve motor execution and planning (Hauk et al., 2004; Desai et al., 2010, 2011, Desai et al., 2013). Patient studies have also shown that degradation of these regions due to stroke or movement disorders (e.g., Parkinson’s disease) has been associated with greater impairments to action-related verb and noun comprehension compared to non-action counterparts (Fernandino et al., 2013a,b; Desai et al., 2015; Johari et al., 2019; Riccardi et al., 2019, 2020).

Non-invasive brain stimulation has also been used to investigate the role of action-related brain areas in lexical semantic processing. However, most of these studies have focused on the effects of M1 stimulation on action verbs specifically, with a relative lack of investigations focusing on manipulable noun comprehension or stimulation of higher-order motor planning areas such as aIPL. For example, transcranial magnetic stimulation (TMS) of the left M1 affected the processing of action verbs relative to abstract verbs when measured by an explicitly semantic task (concreteness judgment; Vukovic et al., 2017). Specific somatotopic representation of effector-related action verbs has also been demonstrated with TMS, with stimulation over the left primary motor hand or leg area affecting lexical decision response times for arm- and leg-related verbs, respectively (Pulvermuller et al., 2005). Transcranial direct current stimulation (tDCS) over M1 and pre-motor cortex enhanced the learning of novel action verbs and accelerate response time for existing action verbs compared to abstract verbs in healthy subjects (Liuzzi et al., 2010; Gijssels et al., 2018). tDCS of left M1 improved recall of sentences with action verbs compared to non-action sentences (Vitale et al., 2021). Moreover, the application of tDCS over left M1 facilitated lexical retrieval of action verbs relative to object words in post-stroke aphasia (Branscheidt et al., 2018). In a recent study, we demonstrated that high definition tDCS of hand motor area (HMA) enhanced the processing of sentences with literal and figurative action verbs compared to sentences with visual verbs (Johari et al., 2021).

Regarding aIPL, the few brain stimulation studies concerning embodied semantics have largely focused on manipulable object comprehension, but not action verbs. Using repetitive TMS, Ishibashi et al. (2011) found that aIPL stimulation resulted in longer response times for explicit manipulation-related semantic judgments of nouns but not for function-related judgments (see also Ishibashi et al., 2018). Similarly, a tDCS investigation revealed that aIPL stimulation modulated response times for semantic relation judgments for tools, but only when the relations were based on functional similarity (De Bellis et al., 2020). Almeida et al. (2017) found that tDCS stimulation of aIPL significantly modulated neural responses in aIPL for tools, but not for faces, animals, or places, as measured by post-tDCS functional neuroimaging. Finally, Ward et al. (2022) found that repetitive TMS of the left intraparietal sulcus (proximal to the aIPL) modulated transitive, but not intransitive, action picture naming performance.

In sum, several gaps remain in the brain stimulation literature pertaining to HMA and aIPL contribution to lexical semantic processing. First, while the effects of HMA stimulation on action verb processing have been shown, it is unclear if these effects will also be seen for manipulable nouns, which also have associated action-related conceptual features (e.g., associated actions related to tool use). Conversely, aIPL stimulation has been shown to affect manipulable object processing during explicit semantic judgments, but its functional contribution (or lack thereof) to action verb processing requires investigation, especially considering the aIPL’s well-established role in action planning and execution (Jubault et al., 2007; Chong et al., 2008; Gallivan et al., 2016). Finally, the lexical semantic system can be tested using a variety of tasks that vary in the depth of semantic processing involved. Semantically implicit tasks such as simple lexical decisions can be successfully completed without explicitly accessing deeper conceptual properties of the words. Explicit tasks, such as semantic similarity judgments, require access to deeper semantic features for successful completion. The majority of previous studies use either an implicit or explicit task, with very few probing multiple levels of processing depth within the same group of participants. By varying the implicit/explicit task demands, one can investigate the automaticity and effects of task demands for HMA or aIPL involvement in the comprehension of action verbs and manipulable nouns. The present study addresses these gaps by examining the effect of high definition tDCS (HD-tDCS) over left HMA and aIPL on action-related verb and noun processing at multiple semantic depths.

HD-tDCS is a relatively recent neurostimulation technique that delivers electrical current with several small electrodes in a pre-defined configuration that provides more precise spatial resolution compared to conventional tDCS (Garnett and den Ouden, 2015; Ho et al., 2016; Rawji et al., 2018). HD-tDCS offers a longer post-stimulation effect (more than 2 h; see Kuo et al., 2013) compared to conventional tDCS which allows to run multiple tasks in one session. Here, we investigated the effect of stimulation of HMA and aIPL on action verbs and manipulable nouns during tasks that probe three semantic loads: (1) subliminal (masked priming during lexical decision); (2) implicit (lexical decision), and (3) explicit (semantic similarity judgment). At each level of semantic load, we compared the effect of stimulation on the processing of action vs. non-action verbs and manipulable vs. non-manipulable nouns. Based on the findings of previous tDCS studies, we hypothesized that the stimulation of both left HMA and aIPL would facilitate the accuracy and response time of action-related words relative to non-action words at one or more levels of semantic load. If these regions have a fundamental role in representing meaning of action-related words that are activated automatically, then implicit tasks should show an effect of stimulation. On the other hand, if these regions play a role only in the context of explicit task demands, then an effect would be seen only in the similarity judgment task. Moreover, it is possible that effects of HMA stimulation would be marginally stronger for action verbs due to their direct-action associations, while aIPL would show a preference for manipulable nouns due to aIPL being especially involved in higher-order action planning and object processing (Orban and Caruana, 2014; Orban et al., 2021).

## Methods

### Subjects

Forty-two healthy right-handed volunteers participated in the study (27 females; mean age 21.3, SD 2.7, range 18–32 years). They had normal or corrected-to-normal vision and hearing and reported no history of neurological, psychiatric, speech, or language disorders. Written consent was obtained from all participants, and they received monetary compensation or extra course credit for their participation. Half of the participants were randomly assigned to the HMA experiment, and half were assigned to the aIPL experiment (see “Procedure” Section below). All study procedures were approved by the University of South Carolina Institutional Review Board.

### Materials

The lexical decision task consisted of 104 verbs, 104 nouns and 108 nonwords. Nonwords were selected from the English Lexicon Project (ELP) database^1^ (Balota et al., 2007), such that both verbs and nouns were matched with nonwords in a number of letters, bigram frequency, and lexical decision accuracy. Half of the verbs were related to voluntary hand/arm actions (e.g., *to tie*, *to knot*), and the others referred to non-action visual or cognitive verbs (e.g., *to view*, *to perceive*). For nouns, half were physically manipulable (e.g., *the ball*, *the pen*) and the other half were comparatively non-manipulable (e.g., *the cabin*, *the roof*). Action and non-action verbs as well as manipulate and non-manipulable nouns were matched in a number of letters, phonemes, and syllables, as well as bigram frequency, number of orthographic and phonological neighbors, and lemma frequency (Table 1). The two conditions within both verbs and nouns were also matched in familiarity, imageability, semantic diversity (Hoffman et al., 2011), mean naming response time (RT) and in lexical decision RT and accuracy, according to the ELP database. Body-object interaction (BOI) ratings (Tillotson et al., 2008; Pexman et al., 2019), which assess how easily the human body can interact with a word’s referent, were significantly different for manipulable compared to non-manipulable nouns, as expected.

**Table 1:** Characteristics of words used in the lexical decision task.

	**Verbs**					**Nouns**				
	**Action**	**SD**	**Non-action**	**SD**	**T-test p**	**Manip**	**SD**	**Non-manip**	**SD**	**T-test p**
NLett	4.74	1.07	5.02	1.35	0.24	5.74	1.65	5.72	1.71	0.95
NPhon	3.70	0.82	3.89	1.08	0.32	4.52	1.34	4.63	1.57	0.69
NSyll	1.13	0.34	1.20	0.45	0.34	1.74	0.68	1.74	0.65	1
Log F	1.25	0.61	1.19	0.66	0.63	1.08	0.55	1.14	0.47	0.55
LD RT	625.13	60.80	641.08	74.88	0.23	639.14	67.69	649.85	51.33	0.36
LD ACC	0.96	0.06	0.96	0.04	0.56	0.97	0.05	0.96	0.04	0.71
Naming RT	620.22	51.04	626.06	51.47	0.55	623.86	52.90	623.56	47.05	0.98
Bigram F	1,587.84	807.84	1,572.70	719.82	0.92	1,716.12	750.34	1,572.71	693.16	0.31
SemD	1.63	0.33	1.67	0.24	0.47	1.58	0.22	1.53	0.20	0.18
Orth N	5.48	4.92	5.65	4.70	0.86	3.59	4.72	2.94	4.19	0.452
Phon N	14.43	11.09	11.96	10.27	0.23	9.09	10.77	7.28	7.28	0.359
Familiarity	531.34	50.21	528.71	55.80	0.84	528.56	57.96	513.29	54.73	0.207
Imageability	512.59	60.82	486.38	71.15	0.11	584.31	35.37	590.73	22.76	0.313
BOI	-	c-	-	c-	-	5.27	0.59	3.65	1.06	<0.001

SemD, semantic diversity; BOI, body-object interaction rating; LD, lexical decision from ELP database.

The semantic similarity judgment task consisted of 48 action verbs, 48 non-action cognitive or visual verbs, 51 manipulable, and 51comparatively non-manipulable nouns. Each set was organized into triplets (32 for verbs, 34 for nouns), such that in each triplet, two of the words had similar meanings. Each verb and noun was used in multiple triplets. The action and non-action conditions within verbs and nouns were matched in a number of letters, phonemes, syllables, and orthographic and phonological neighbors, lemma frequency, as well as in mean naming, and LD RT according to the ELP (Table 2). Similar to the LD task, manipulable and non-manipulable nouns differed in BOI ratings. All experimental parameters (e.g., timing of trials and randomizing) were administered with Eprime (Psychology Software Tools, Pittsburgh, PA). For any given subject, there were no overlaps between stimuli used for active and sham stimulations for both LD and SSJ tasks.

**Table 2:** Characteristics of the words used in the semantic similarity judgment task.

	**Verbs**					**Nouns**				
	**Action**	**SD**	**Non-action**	**SD**	**T-test p**	**Manip**	**SD**	**Non-manip**	**SD**	**T-test p**
NLett	5.07	1.05	5.09	0.81	0.89	5.62	1.64	5.61	1.64	1
NPhon	4.03	0.79	4.08	0.86	0.78	4.44	1.34	4.53	1.57	0.51
NSyll	1.28	0.42	1.35	0.35	0.35	1.57	0.60	1.64	0.75	0.36
Log F	1.16	0.40	1.15	0.37	0.83	1.02	0.56	1.09	0.52	0.22
LD RT	658.06	44.89	645.98	41.18	0.16	658.31	70.98	650.43	62.83	0.31
Naming RT	630.37	39.93	627.90	31.86	0.73	634.61	62.61	628.80	53.08	0.36
Bigram F	1,577.68	456.45	1,708.22	379.43	0.12	1,649.84	731.30	1,621.85	779.34	0.77
SemD	1.68	0.16	1.72	0.15	0.25	1.52	0.23	1.49	0.21	0.41
BOI	-	c-	-	c-	-	5.16	0.95	3.76	1.31	<0.001

### Procedure

**Figure 1:**
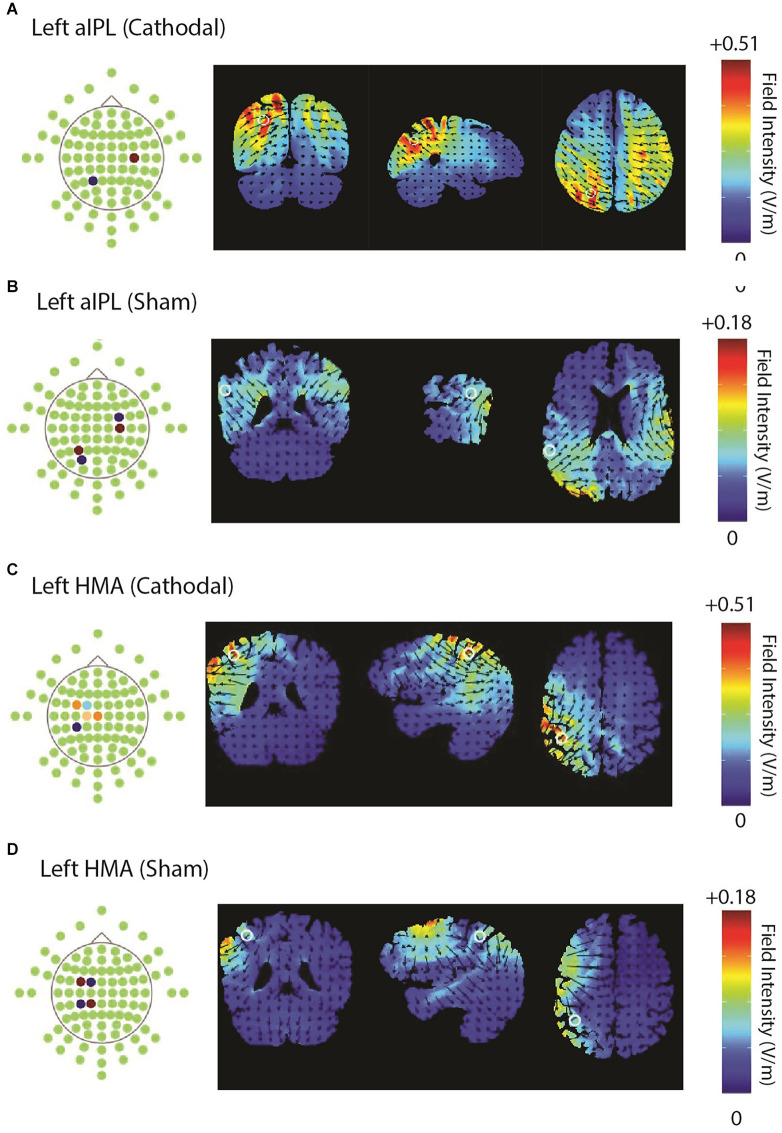
Illustrates the electrode configurations and modeled flow of current for both active and sham stimulations over aIPL panel **(A,B)** and HMA panel **(C,D)**.

Two experiments were conducted, targeting the left hand motor cortex (HMA) and left inferior parietal lobule (aIPL) sites respectively. Stimuli, procedures, and analyses were identical for both experiments, except for the stimulation site. Subjects were randomly assigned to one of the two experiments, and received active and sham stimulations for 20 min, applied with an M×N HD-tDCS Stimulator (Soterix Medical Inc., NY, USA). The configuration of the electrodes and their corresponding current intensities are shown in Table 3 for HMA and aIPL. HD-Explore and HD-Target software (Soterix Medical Inc., NY, USA) were used to compute electrode configurations (locations and intensities) based on the current simulation. Figure 1 shows the electrode locations and anatomical position for HMA (MNI: *x* = −38, *y* = −31, *z* = 52) and aIPL (MNI: *x* = −57, *y* = −36, *z* = 34) as well as the modeled pattern of current flow intensity for active and sham HD-tDCS for both stimulation targets.

**Table 3:** Electrode configurations.

**Stimulation target site**	**Stimulation type**	**Electrode configuration (MCN system)**	**Resulting intensity at target coordinates in L-aIPL**	**Resulting intensity at target coordinates in L-HMA**
**aIPL**	Active	C4 (+2.0 mA), P3 (−2.0 mA)	0.57 V/m	0.33 V/m
	Sham	C4 (+1.0 mA), FC4 (−1.0 mA), P3 (+1.0 mA), PO3 (−1.0 mA)	0.06 V/m	0.05 V/m
**HMA**	Active	Cz (+0.88 mA), FC3 (+0.84 mA), C1 (+0.14 mA), Cp3 (−1.85 mA), FC1 (−0.01 mA)	0.33 V/m	0.54 V/m
	Sham	FC3 (+1.0 mA), FC1 (−1.0 mA), CP1 (+1.0 mA), CP3 (−1.0 mA)	0.07 V/m	0.05 V/m

A nominally cathodal stimulation was used. However, we note that for HD-tDCS configurations, the traditional tDCS notions of cathodal/inhibitory and anodal/excitatory stimulations are not clearly applicable (Garnett and den Ouden, 2015). To administer HD-tDCS, a standard 10–20 EEG cap (Easy-Cap GmbH, Germany) was placed on the subject’s head, with the Cz position midway between inion and nasion, and between the two mastoids. The control stimulation was an “active sham”, where stimulation was administered for the entire 20 min but in a montage where the current was modeled to bypass the cortex and have minimal stimulation of the target area (Davis et al., 2013; Richardson et al., 2014; Garnett and den Ouden, 2015). Electrodes were placed in proximal pairs so that the current was flowing in and out at adjacent electrodes. Particularly with high-definition multiple-electrode configurations, the sham method often used in traditional tDCS is to ramp up and then ramp down the current to induce the sensation of stimulation onset, but this may not be sufficient to fully neutralize differences in sensitivity between active and sham stimulation (Richardson et al., 2014). Even though excitation/inhibition of neurons under the sham electrodes cannot be completely ruled out with an active sham used here, this was not expected to substantially affect task responses (Ambrus et al., 2012; Kessler et al., 2012).

The experiments were conducted in a sound attenuated booth. Stimuli were displayed on a screen and presented in a pseudo-random order. During the neurostimulation session, subjects performed a non-language distraction task (silently working on a jigsaw puzzle) and started doing the tasks immediately after the stimulation finished. The order of the stimulation sessions was counterbalanced between the subjects. Half of the subjects received active stimulation in the first session followed by sham stimulation in the second session. The rest of the subjects received sham and active stimulations in the first and second sessions, respectively. Subjects were not aware about the type of stimulation they were receiving in each session.

**Figure 2:**
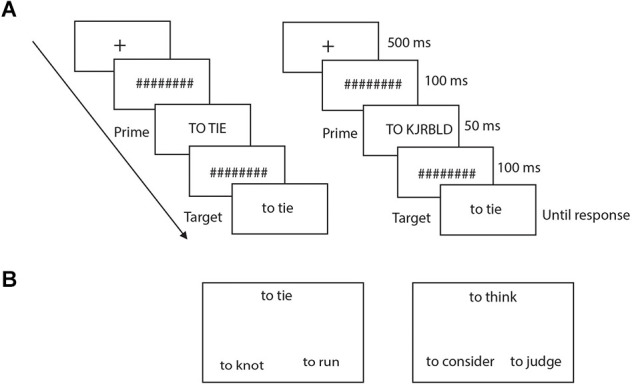
**(A)** Illustrates the sequence for each trial of the LD task for both identity prime (left panel) and random prime (right panel). **(B)** Displays of an action (left) and of an abstract (right) trial of the SSJ task.

Following stimulation, subjects performed lexical decision (LD) and semantic similarity judgment (SSJ) tasks, the order of which was counterbalanced between subjects. For LD, each trial started with a fixation cross that appeared in the center of the screen for 500 ms (Figure 2A). The fixation was followed by a series of eight hash marks, presented for 100 ms, followed by the prime stimulus (50 ms), another series of eight hash marks (100 ms), and the target stimulus, which remained on the screen until the participant made a response. Each word and pseudoword was presented with the word “to” for verbs and “the” for nouns to their left. The prime was either the same as the target word or a consonant string, also preceded by the word “to” or “the” for verbs and nouns. Prime words were presented in upper case font and targets in lower case font to make them perceptually distinct. The priming manipulation was counterbalanced across subjects, so that each word was primed by the capitalized target and by the consonant string an equal number of times. For example, half of the subjects saw the items “to tie” and “the bell” primed with capitalized targets and the items “to knot” and “the pen” primed with consonant strings, while the remaining participants saw the items “to tie” and “the bell” primed with consonant strings and the items “to knot” and “the pen” primed with capitalized targets. Subjects were asked to judge as quickly and as accurately as possible whether the target was a word or not by pressing one of two response keys with their right hand. The position of the two response keys (left-right to indicate word-nonword) was counterbalanced across subjects. Subjects underwent 10 practice trials before starting the actual tasks.

For SSJ, three verbs were presented simultaneously in a triangular arrangement (Figure 2B). Each stimulus was presented with the word “to” and “the” to their left, respectively for verbs and nouns. Subjects were asked to judge which of the two words on the bottom had the most similar meaning to the one on top and indicate their response as quickly and accurately as possible by pressing one of two response keys. The words remained on the screen until the subjects made a response. There were 48 trials for verbs and 52 for nouns, divided equally between action and non-action verbs as well as between manipulable and non-manipulable nouns. The position of the two bottom words on the screen (left or right) was counterbalanced across subjects. Subjects received practice trials before the actual experiment. Note, for each subject, there were no overlaps between stimuli used for active and sham stimulations for both LD and SSJ tasks.

### Data analysis

For each subject, the accuracy was calculated based on the total number of correct responses for each task. The reaction time (RT) for each task was obtained by the time difference between stimuli presentations and subjects’ response. Trials with ± 3 SD away from the mean RT were considered outliers and were excluded from statistical analysis. The average outlier rate was <3% across participants. Then to minimize variability across trials, raw RT was converted to z-score for each task. Hereafter, RT refers to z-scores of response time. As mentioned in the introduction, our hypotheses primarily concerned the interaction of stimulation and tasks. We predicted that stimulation of left aIPL and HMA would significantly affect the accuracy and RT of action verbs and manipulable nouns compared to non-action verbs and non-manipulable nouns, respectively. In order to directly test these hypotheses, for each subject, we calculated the net RT and net accuracy by subtracting the RTs and correct responses of sham stimulation from active stimulation for each task. Therefore, a negative net RT or positive net accuracy would indicate stimulation-related task facilitation for a given condition (e.g., faster RT and higher accuracy during stimulation compared to sham). Additional analysis was performed within the LD task to examine the effect of stimulation on RT and the accuracy of stimuli preceded with identity vs. random primes. The net RT and net accuracy for priming were obtained by subtracting random prime from identity prime, and then the net priming effect was computed by subtracting net RT and net accuracy of sham from active. Therefore, a positive net priming RT or negative net priming accuracy value would indicate stimulation-related facilitation of the identity priming effect compared to sham. For statistical analysis, one tailed t-tests were used to examine the following contrasts: (1) action vs. non-action verbs; (2) manipulable vs. non-manipulable nouns; with net accuracy, net RT, and net priming as dependent variables. As discussed in Johari et al. (2021) and Fernandino et al. (2013) the interactions in repeated measure ANOVA are equivalent to computing a “net RT” (cathodal RT-sham RT) for each condition, and comparing conditions with a t-test, with the difference that directional testing is possible for t-tests, while it is not for ANOVAs (Howell, 2012; Fernandino et al., 2013b). Since our analyses were hypothesis-driven with planned comparisons, we did not apply corrections for multiple comparisons.

We were also interested in directly comparing HMA and IPL, with the exploratory hypothesis that HMA is involved in explicit semantic tasks (SSJ), while aIPL is important for both implicit (LD) and explicit tasks. Repeated measure ANOVA was used to examine this exploratory hypothesis. Cohen’s d (d) and partial eta squared ($\eta_{p}^{2}$) were reported for one tailed t-tests and ANOVA respectively as a measure of effect size. All statistical analyses were performed using Statsmodels in Python^2^. Depending on the task and strategy adopted by the participants, the effects could arise either in speed, in accuracy, or both, due to the speed-accuracy tradeoff (Standage et al., 2014). Hence, we do not make specific predictions about effects arising in RT or accuracy but expect that they could arise in either.

## Results

### Effects of HD-tDCS on HMA

**Figure 3:**
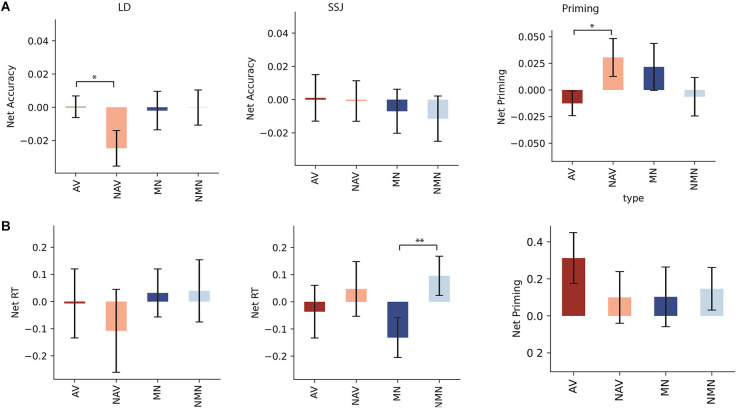
**(A)** Net accuracy and **(B)** net RT following HMA stimulation for LD, SSJ, and priming. Net accuracy refers to the difference between accuracy following active and sham stimulations. Net RT refers to the difference in z score of response time between active and sham stimulation. Significant differences were marked with asterisk signs (**for *p* < 0.01 and *for *p* < 0.05). Task abbreviations: AV, action verb; NAV, non-action verbs; MN, Manipulable nouns; NMN, Nonmanipulable nouns.

Lexical decision: Figure 3 shows the means of net accuracy (Figure 3A) and net RT (Figure 3B) for each task following stimulation of HMA. HMA stimulation significantly decreased net accuracy for non-action verbs compared to action verb in LD (*t*_(20)_ = 2.07, *p* = 0.025, *d* = 0.63). Action and non-action verbs did not significantly differ in net RT (*t*_(20)_ = 1.022, *p* = 0.16, *d* = 0.16). Manipulable and non-manipulable nouns did not significantly differ in net accuracy (*t*_(20)_ = −0.19, *p* = 0.42, *d* = −0.036) or net RT (*t*_(20)_ = −0.08, *p* = 0.47, *d* = −0.016).

Priming: Net priming results indicated that simulation improved net accuracy of primed action verbs compared to primed non-action verbs (*t*_(20)_ = −1.91, *p* = 0.034, *d* = −0.64), whereas there was no such effect for manipulable vs. non-manipulable nouns (*t*_(20)_ = 0.98, *p* = 0.17, *d* = 0.30). RT analysis for net priming did not reveal significant differences between action and non-action conditions for either nouns (*t*_(20)_ = −0.22, *p* = 0.41, *d* = −0.069) or verbs (*t*_(20)_ = 1.13, *p* = 0.14, *d* = 0.34).

SSJ: Stimulation of HMA significantly accelerated SSJ RT for manipulable compared to non-manipulable nouns (*t*_(20)_ = −3.48, *p* = 0.001, *d* = −0.68), but not for action compared to non-action verbs (*t*_(20)_ = −0.79, *p* = 0.22, *d* = −0.18). Net accuracy analysis did not reveal significant differences between action and non-action conditions for either nouns (*t*_(20)_ = 0.26, *p* = 0.40, *d* = 0.07) or verbs (*t*_(20)_ = 0.13, *p* = 0.45, *d* = 0.03).

### Effects of HD-tDCS of aIPL

**Figure 4:**
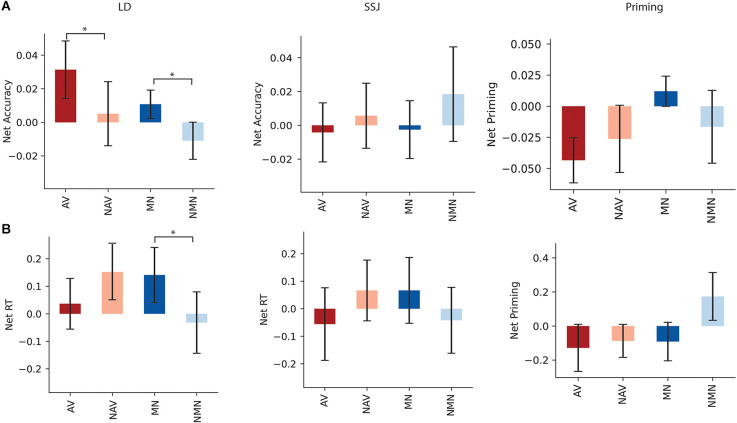
Panels **(A,B)** show the net accuracy and net RT following aIPL stimulation for LD, SSJ, and priming. Net accuracy refers to the difference between accuracy following active and sham stimulations. Net RT refers to the difference in z score of response time between active and sham stimulation. Significant differences were marked with asterisk signs (*p* < 0.05). Tasks abbreviations: AV, action verbs; NAV, Non-action verbs; MN, Manipulable nouns; NMN, Non-manipulable nouns.

Lexical Decision: The mean net accuracy and net RTs following stimulation of aIPL are shown in Figures 4A,B respectively. Stimulation of aIPL significantly improved net accuracy of action verbs compared to non-action verbs in LD (*t*_(20)_ = 2.17, *p* = 0.021, *d* = 0.32), and net accuracy of manipulable nouns relative to non-manipulable nouns (*t*_(20)_ = 2.22, *p* = 0.02, *d* = 0.49). Net RT indicated stimulation of aIPL slowed RTs for manipulable nouns compared to non-manipulable nouns in LD (*t*_(20)_ = 1.75, *p* = 0.047, *d* = 0.356), and no significant RT differences were found for action compared to non-action verbs (*t*_(20)_ = −1.05, *p* = 0.15, *d* = −0.26).

Priming: There was a trending difference between RT of net priming for manipulable vs. non-manipulable (*t*_(20)_ = −1.33, *p* = 0.10, *d* = −0.45), with faster reaction time following stimulation of left aIPL for primed manipulable vs. primed non-manipulable nouns. There was no significant difference between RT of net priming for action vs. non action (*t*_(20)_ = −0.22, *p* = 0.41, *d* = −0.08), and there were no significant differences between the accuracy of net priming for action and non-action verbs (*t*_(20)_ = −0.76, *p* = 0.23, *d* = −0.17), nor manipulable and non-manipulable nouns (*t*_(20)_ = 0.99, *p* = 0.16, *d* = 0.30).

SSJ: There were no significant differences in action compared to non-action conditions for either verbs or nouns in RT (*t*_(20)_ = 1.03, *p* = 0.16, *d* = 0.20; *t*_(20)_ = −0.96, *p* = 0.17, *d* = −0.22) or accuracy (*t*_(20)_ = −0.35, *p* = 0.36, *d* = −0.11; (*t*_(20)_ = −0.58, *p* = 0.28, *d* = −0.20).

### HMA vs. aIPL interaction

We conducted an exploratory analysis to examine the effects of stimulation location on action words, and on semantic task explicitness (SSJ vs. LD), collapsed across nouns and verbs. For action vs. non-action words, no significant interactions [site (HMA, aIPL) × word type (action, non-action)] were found for either LD, Priming, or SSJ (all *p* > 0.1), with the following exception. A significant interaction for manipulable vs. noun-manipulable noun SSJ RT was found (*F*_(1,40)_ = 7.36, *p* = 0.009, $\eta_{p}^{2}$ = 0.16). A trend for manipulable vs. non-manipulable noun LD accuracy was also found (*F*_(1,40)_ = 3.91, *p* = 0.06, $\eta_{p}^{2}$ = 0.09).

For explicit vs. implicit tasks collapsing across nouns and verbs, no interactions were found either in RT or accuracy (both *p* > 0.3).

## Discussion

The present study investigated the effects of HD-tDCS centered over the HMA and aIPL on tasks that probed action-related lexical semantic processing at three levels of semantic depth: subliminal (masked priming during LD), implicit (LD), and explicit (SSJ). Broadly, we found that stimulation of these primary (HMA) and higher-order (aIPL) motor areas resulted in relative facilitation of action-related language processing compared to non-action, aligning with findings from previous brain stimulation studies (Liuzzi et al., 2010; Branscheidt et al., 2018; Gijssels et al., 2018; Johari et al., 2021; Vitale et al., 2021). The majority of significant findings in the present study had a medium effect size, and few findings had small effect size as measured by Cohen’s d and partial eta squared ($\eta_{p}^{2}$). The current study was unique in its use of multiple stimulation sites, grammatical classes, and semantic loads, allowing for a finer-grained investigation of the specific roles of HMA and aIPL in lexical semantic processing of action-related words, which will be discussed below.

### HMA

Stimulation of HMA was associated with relative facilitation of action verb compared to non-action verb LD at both subliminal and implicit semantic levels. These results align with previous studies demonstrating the effects of HMA stimulation on action verb processing measured by implicitly semantic tasks (e.g., LD; Pulvermuller et al., 2005; Branscheidt et al., 2018; Vukovic and Shtyrov, 2019), and demonstrate that these HMA faciliatory effects extend to a subliminal level of semantic processing for verbs (i.e., masked priming). Lexical semantic representations have been shown to be context- and task-dependent (van Dam et al., 2010, 2012; Yee and Thompson-Schill, 2016), with some studies suggesting that distributed action-perception areas may only be involved in word representation when a deeper semantic analysis is encouraged (Meteyard et al., 2012). As such, effects seen at subliminal and implicit semantic levels are often interpreted as strong evidence for the functional contribution of action-perception brain areas to lexical semantic representation, as these tasks do not explicitly require the retrieval of semantic information. The present findings, when considered with the previously discussed studies concerning HMA stimulation and action verbs at sub-explicit semantic loads, suggest that the HMA is specifically and functionally involved in the representation of action verbs at relatively basic levels of comprehension. Considering the present study’s close matching of action and non-action verb stimuli across a variety of psycholinguistic variables (Tables s 2, 3), this representation can be interpreted as relating to the salience of motor information to action verbs, and it suggests that this motor-related information is: (1) partially represented within HMA, and (2) is important for action verb processing even when it does not need to be accessed explicitly.

At the explicit level, stimulation of HMA facilitated manipulable compared to non-manipulable noun semantic judgments, but this effect was not seen for action compared to non-action verbs. To our knowledge, this study is one of the first to explore the effects of HMA stimulation on manipulable noun comprehension, as most prior studies focused on HMA and action verbs (Vukovic et al., 2017; Branscheidt et al., 2018; Gijssels et al., 2018; Vukovic and Shtyrov, 2019). Considering the negative results for manipulable nouns at sub-explicit levels, the finding of relative facilitation for manipulable nouns during an explicit semantic task suggests that motor-related representational content stored within HMA may not be important for manipulable noun processing unless a deeper semantic analysis is required. Information related to manipulable nouns may be partially represented by HMA, but this information is only accessed in certain task contexts. In the present study, “semantic similarity” for manipulable nouns was based largely on a mixture of shared manipulation-related, functional, and structural properties of the nouns. That is, the target and the correct response tended to have similar appearances, be manipulated using similar hand motions, and serve similar purposes (e.g., the shovel and the spade). It is therefore unclear exactly what features are stored within the HMA that are accessed during explicit manipulable noun comprehension. Considering action verb results from the present study, as well as the previously discussed literature, a reasonable hypothesis is that the informational content that is being accessed during explicit manipulable noun semantics is either related to actual motoric similarities in their use (e.g., shovel and spade are manipulated using the same hand/arm motions) or related to functional similarities that are defined by action and event understanding (e.g., we know that shovel and spade are both used to dig). Future studies investigating HMA in manipulable noun comprehension should seek to adjudicate between these possible explanations.

The negative results for action verbs at the explicit level were somewhat surprising. If stimulation facilitates action verb processing at sub-explicit levels, why would it not also affect explicit action verb semantics? One explanation is an interaction between the SSJ task demands and the somewhat coarse spatial resolution of tDCS, at least compared to TMS. While the SSJ task has deeper semantic requirements than the LD, it is also more demanding at a cognitive/executive control level. For a successful trial, participants must read and comprehend all three words, compare their meaning, make a decision to choose the correct response, and ignore the distractor word. These control-level processes are shared for both the action and non-action verb conditions. While HMA stimulation was centered on HMA, it also extends somewhat to the frontal and temporoparietal regions (Figure 1C). These frontal and temporoparietal regions may serve processes that underly cognitive control or other operations shared by action and non-action verbs in this task context (Kayser et al., 2010; Rahnev et al., 2016), and thus the stimulation may have affected both action and non-action verb conditions similarly with respect to these control processes.

### aIPL

At sub-explicit semantic loads, aIPL stimulation was associated with a trending facilitatory priming effect for manipulable nouns relative to non-manipulable. It also significantly facilitated manipulable noun and action verb LD accuracy relative to non-action control conditions, while at the same time slowing manipulable noun RT. No significant effects were seen for verbs or nouns at the explicit level. The present results broadly align with prior studies showing that aIPL stimulation modulates behavioral and neural responses to manipulable objects/tools (Ishibashi et al., 2011, 2018; Almeida et al., 2017). Further, the finding of facilitated action verb comprehension adds to the body of research, as very few prior studies have directly investigated the effects of aIPL stimulation on action verb comprehension.

aIPL has been implicated as a higher-order motor area that is involved in action planning and execution, especially related to tool use (Jubault et al., 2007; Chong et al., 2008; Gallivan et al., 2016). Neuroimaging and brain stimulation studies have provided evidence that aIPL also serves the processing of manipulable nouns, suggesting that it is an area of overlap between actual manipulation of objects and manipulable object lexical semantics (Chong et al., 2008; Ishibashi et al., 2018; De Bellis et al., 2020). The present results, of stimulation-related facilitation of priming RT and LD accuracy compared to closely matched non-manipulable nouns, support this hypothesis. These effects at sub-explicit loads are of particular interest, considering that prior brain stimulation studies of aIPL and manipulable object knowledge have largely used explicit semantic judgment tasks (Ishibashi et al., 2011, 2018; De Bellis et al., 2020). Effects at sub-explicit loads suggest that the aIPL supports the processing of manipulable nouns even when a deeper semantic analysis is not required, perhaps due to tool-use representations stored within aIPL being particularly salient for manipulable noun understanding (i.e., tool-use information helps represent manipulable nouns even in shallow contexts). The relative stimulation-related slowing of manipulable noun LD RT compared to non-manipulable nouns is somewhat surprising, considering that all other effects observed in the current study were faciliatory. One possible explanation is stimulation-related speed-accuracy trade-off for manipulable nouns specifically. That is, perhaps aIPL stimulation facilitated deeper semantic analysis for manipulable nouns than is necessary to successfully complete the LD task, resulting in high accuracies for manipulable nouns with simultaneously slower RTs.

Similar to manipulable noun facilitation, stimulation-related facilitation of action verb LD accuracy suggests that the aIPL plays an important role in action-related language processing. This finding is particularly novel, as prior brain stimulation studies of semantics and the aIPL have focused specifically on manipulable objects. Considering that this action verb effect was seen in an implicit task, as well as in the HMA stimulation, it supports hypotheses suggesting that both primary and higher-order action areas functionally contribute to lexical semantic representations of action verbs even when task demands do not explicitly orient attention to action-related semantic features.

Negative results for the SSJ task are again somewhat surprising. aIPL is considered a “higher-order” action area, as opposed to primary, with the implication being that it contains somewhat abstracted information related to object-oriented action planning and execution as opposed to more direct motor representations housed in primary cortices (Jubault et al., 2007; Chong et al., 2008). However, the aIPL is also directly adjacent to areas related to executive function, such as those related to attention and eye movements (e.g., intraparietal sulcus; Geng and Mangun, 2009; Gillebert et al., 2011; Choi et al., 2014), which would be expected to be involved in both action and non-action related language processing during an explicit semantic task that involves reading three words and comparing pairwise meanings. Just as non-focal HMA stimulation potentially affects frontal regions involved in executive function, the parietal components of the fronto-parietal network may be affected by the low-spatial-resolution stimulation of aIPL. Hence, condition-specific effects may be difficult to observe in executively demanding tasks such as SSJ when a larger parietal area is stimulated.

Direct comparison of HMA and aIPL stimulation did not reveal significant differences associated with stimulation location, except for manipulable vs. non-manipulable nouns. These results suggest that HMA and aIPL are both involved in action semantics, regardless of task demands. Our preliminary hypothesis, that the role of two areas depends on the explicitness of the task, was not supported. Ward et al. (2022) found that TMS stimulation near aIPL affected transitive, but not intransitive action verb naming. They also found that the ventral premotor stimulation affected both transitive and intransitive picture naming. This result, combined with our finding for manipulable nouns, suggests that aIPL is crucial for actions involving visuo-motor coordination. Hand-object interaction and visual guidance for action are necessary for both manipulation of objects and for performing transitive actions (e.g., throwing a ball, picking up a pencil), but not for performing intransitive or non-object-oriented actions (e.g., laughing, jumping). Primary motor areas, on the other hand, are involved in both transitive and intransitive actions, playing a more general role in action semantics. This involvement is automatic, in that it is seen even when the task does not require explicit access to action semantic features of words.

### Limitations

The spatial resolution of HD-tDCS can be a limitation when investigating a brain area that is adjacent to domain-general regions, which is the case for both HMA and aIPL. Leaking stimulation to those regions can impact performance on a variety of tasks, masking the ability to detect task-specific effects. This limitation can be exacerbated by using tasks that rely on a variety of cognitive operations (e.g., the SSJ task), as the overlapping cognitive processes between the conditions of interest (e.g., action vs. non-action language) increase as general cognitive demands increase. Techniques with greater spatial resolution such as TMS may address this limitation in future studies. Another limitation is that, in the SSJ task, “semantic similarity” for nouns relied on a mixture of manipulation-related (e.g., how does one typically manipulate this object), functional (e.g., what is this object used for), and structural (e.g., what does this object look like) properties. There is evidence that these types of knowledge may be at least partially dissociable in the brain (Buxbaum and Saffran, 2002; Buxbaum and Kalenine, 2010; Ishibashi et al., 2011, 2018). Future studies could manipulate these types of knowledge in order to investigate the anatomical organization of manipulable noun representation.

## Conclusion

The present study investigated the effects of HD-tDCS centered over the HMA and aIPL on tasks that probed action-related lexical semantic processing at three levels of semantic depth: subliminal (masked priming during LD), implicit (LD), and explicit (SSJ). Broadly, we found that stimulation of these primary (HMA) and higher-order (aIPL) motor areas resulted in relative facilitation of action-related language processing compared to non-action. HMA stimulation facilitated action verb processing in subliminal and implicit task contexts, suggesting that HMA helps represent action verbs even in shallow task contexts. HMA stimulation also facilitated manipulable noun comprehension in an explicit semantic task, suggesting that HMA contributes to manipulable noun comprehension when semantic demands are high. aIPL stimulation facilitated both manipulable noun and action verb processing during an implicit task. Thus, both HMA and aIPL play a functional role in action semantics. HMA plays a general role in the semantics of actions and manipulable objects, while aIPL is important only when visuo-motor coordination is required for the action.

## Data Availability Statement

The raw data supporting the conclusions of this article will be made available by the authors, without undue reservation.

## Ethics Statement

The studies involving human participants were reviewed and approved by University of South Carolina Institutional Review Board. The patients/participants provided their written informed consent to participate in this study.

## Author Contributions

RD designed the study. SM and NR collected the data. MM, KJ, and SM analyzed the data. KJ, NR, and RD wrote the article. All authors contributed to the article and approved the submitted version.

## Funding

This research was supported by NIH/NIDCD grants R01 DC010783, R56DC010783, and R01DC017162 (RD).

## Footnotes

^1^ http://elexicon.wustl.edu

^2^ https://www.statsmodels.org/stable/index.html
